# Mucosal Irregularity on Gastric Contrast Study in a Case of Carmi Syndrome

**DOI:** 10.21699/jns.v6i2.498

**Published:** 2017-04-15

**Authors:** Sudhir Singh, Nitin Pant, JD Rawat, Yadvendra Dheer

**Affiliations:** Department of Pediatric Surgery, King George’s Medical University, Lucknow, India

**Dear Sir**

Carmi syndrome comprises of EB (Epidermolysis Bullosa) and PA (pyloric atresia). We report the radiological finding of mucosal irregularity as seen in upper GI contrast study in the antral region and along the greater curvature in a case of this syndrome. This could act as a clinical tip off for the presence of Carmi syndrome in cases of a solitary PA. It is proposed that in such a case, the PA occurs secondary to an intrauterine complication of EB where sloughing of pyloric mucosa and subsequent fibrosis leads to obstruction of the pyloric canal.

A 2.4-kg, first born, full-term male, from a consanguineous marriage was referred from a primary health centre at day 4 of life. The pregnancy was unregistered. The baby had recurrent non bilious vomiting following feed along with generalised bullae since day one of life. New blisters continued to develop spontaneously. Plain X-ray abdomen showed a single air fluid level due to the residual milk in the stomach (Fig.1A). A water soluble contrast study confirmed a pyloric atresia. It also showed mucosal irregularity in the antral region and along the adjacent greater curvature (Fig.1B). Patient was operated after initial resuscitation and workup. Per operative finding was type 2 PA. There was mucosal thickening and redness noted in gastric mucosa at antral region which corresponded to the findings on contrast study. An end to end gastro duodenostomy was done after excision of atretic segment. The child developed sepsis on the third post-operative day and expired on 5th post-operative day.

Intrauterine sloughing of the mucosa and subsequent scaring may also involve the gastrointestinal, urinary, pulmonary systems and the eye [1]. Surgical intervention for PA in Carmi syndrome is effective only for short- term survival, and the long-term outcome is almost always poor [2]. The antral mucosa in the contrast film is seen elevated and irregular both along the greater curvature as well as to a minor extent along the lesser curvature. This suggests that apart from the atretic region the pathology also extends proximally and the atresia is secondary to mucosal sloughing and subsequent fibrosis rather than failure of recanalization in which case the mucosal outline would have been smooth. The finding mucosal irregularity on a contrast film in a case of solitary pyloric atresia can suggest the clinician to look for/ anticipate involvement of other epithelial surfaces. Since skin lesions may not appear up to 48 hours after birth [3] and death usually is from extensive denudation of skin leading to septicemia, electrolyte imbalance, protein loss, and dehydration, finding mucosal irregularity on a contrast study in a case of pyloric atresia may forewarn an involvement of other epithelial sites especially skin.

**Figure:**
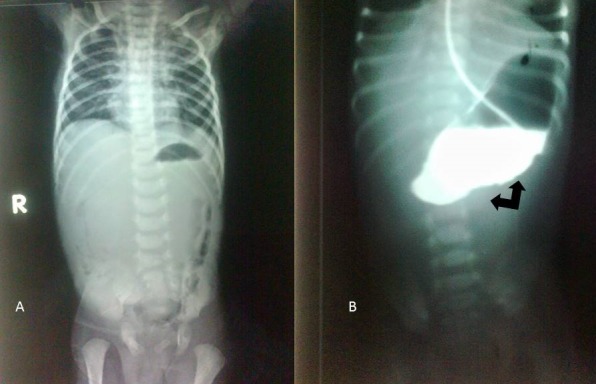
Figure 1: A) Plane abdominal X ray of patient suggestive of pyloric obstruction (some air seen in left colon due to rectal washout done outside). B) Upper GI contrast study. Note the irregular mucosal surface in the antrum along the greater curvature suggesting mucosal involvement (shown by arrow).

## Footnotes

**Source of Support:** Nil

**Conflict of Interest:** None

## References

[1] Chang CH, Perrin EV, Bove KE. Pyloric atresia associated with epidermolysis bullosa: special reference to pathogenesis. Pediatr Pathol. 1983;1:449-57. 10.3109/15513818309025877 6687295

[2] Sarin,YK, Nagdeve NG. Carmi syndrome complicated by pharyngo-esophageal perforation. Indian Pediatrics. 2006;43:61-4. 16465009

[3] Lestringant GG, Akel SR, Qayed KI. The pyloric atresia junctional epidermolysis bullosa syndrome. Report of a case and review of the literature. Arch Dermatol. 1992;128:1083-6. 10.1001/archderm.128.8.1083 1497363

